# Familial Alzheimer’s disease mutations in amyloid protein precursor alter proteolysis by γ-secretase to increase amyloid β-peptides of ≥45 residues

**DOI:** 10.1016/j.jbc.2021.100281

**Published:** 2021-01-12

**Authors:** Sujan Devkota, Todd D. Williams, Michael S. Wolfe

**Affiliations:** 1 Department of Medicinal Chemistry, University of Kansas, Lawrence, Kansas, USA; 2 Mass Spectrometry Laboratory, University of Kansas, Lawrence, Kansas, USA

**Keywords:** intramembrane proteolysis, mass spectrometry, amyloid β-protein (Aβ), amyloid β-protein precursor (APP), pathogenesis, Aβ, amyloid β-protein, AICD, APP intracellular domain, APP, amyloid β-protein precursor, DDM, n-dodecyl-β-D-maltoside, FAD, familial Alzheimer's disease, HEK, human embryonic kidney, LC-MS/MS, liquid chromatography coupled to tandem mass spectrometry, MALDI-TOF, matrix-assisted laser desorption/ionization time-of-flight, MS, mass spectrometry, Q-TOF, quadrupole time-of-flight, TMD, transmembrane domain

## Abstract

Production of amyloid β-protein (Aβ) is carried out by the membrane-embedded γ-secretase complex. Mutations in the transmembrane domain of amyloid β-protein precursor (APP) associated with early-onset familial Alzheimer's disease (FAD) can alter the ratio of aggregation-prone 42-residue Aβ (Aβ42) to 40-residue Aβ (Aβ40). However, APP substrate is proteolyzed processively by γ-secretase along two pathways: Aβ49→Aβ46→Aβ43→Aβ40 and Aβ48→Aβ45→Aβ42→Aβ38. Effects of FAD mutations on each proteolytic step are unknown, largely due to difficulties in detecting and quantifying longer Aβ peptides. To address this, we carried out systematic and quantitative analyses of all tri- and tetrapeptide coproducts from proteolysis of wild-type and 14 FAD-mutant APP substrates by purified γ-secretase. These small peptides, including FAD-mutant forms, were detected by tandem mass spectrometry and quantified by establishing concentration curves for each of 32 standards. APP intracellular domain (AICD) coproducts were quantified by immunoblot, and the ratio of AICD products corresponding to Aβ48 and Aβ49 was determined by mass spectrometry. Levels of individual Aβ peptides were determined by subtracting levels of peptide coproducts associated with degradation from those associated with production. This method was validated for Aβ40 and Aβ42 by specific ELISAs and production of equimolar levels of Aβ and AICD. Not all mutant substrates led to increased Aβ42/40. However, all 14 disease-causing mutations led to inefficient processing of longer forms of Aβ ≥ 45 residues. In addition, the effects of certain mutations provided insight into the mechanism of processive proteolysis: intermediate Aβ peptides apparently remain bound for subsequent trimming and are not released and reassociated.

Cerebral plaques composed of the amyloid β-protein (Aβ) are a defining pathological feature of Alzheimer’s disease (1). Aβ is produced from the amyloid β-protein precursor (APP) through sequential proteolysis, by β-secretase shedding the ectodomain (2) followed by γ-secretase cutting within the transmembrane domain (TMD) of the remnant 99-residue C-terminal fragment (C99) (3). Aβ peptides of 38 to 43 residues are secreted, with the aggregation-prone 42-residue form (Aβ42) being predominantly and disproportionally deposited in AD plaques (4). A pathogenic role for Aβ42 was strongly supported by the discovery of dominant missense mutations in APP and presenilins—the catalytic component of the γ-secretase complex—that cause early-onset familial Alzheimer’s disease (FAD) (5). These mutations were found to elevate the ratio of Aβ42 to Aβ40, thereby increasing Aβ42 aggregation.

Inconsistencies with the hypothesis that Aβ42 is the pathogenic variant in FAD emerged recently with a report on Aβ40 and Aβ42 production from 138 different FAD-mutant forms of the presenilin-1/γ-secretase complex, showing that many disease-causing mutations did not elevate Aβ42/Aβ40 (6). Yet the involvement of Aβ in FAD seems inescapable: after more than 30 years of searching, the only mutations associated with FAD are found in the substrate and enzyme that produces Aβ. Solving this puzzle requires recognition that processing of the APP TMD by the membrane-embedded γ-secretase complex occurs processively. The enzyme first cleaves near the cytosolic end of the APP TMD at the ε site to give either Aβ48 or Aβ49 and release the corresponding APP intracellular domain (AICD) composed of C99 residues 49 to 99 or 50 to 99 (7) (Fig. 1). Carboxypeptidase cleavage generally every three amino acids then produces secreted Aβ peptides along two pathways: Aβ49→Aβ46→Aβ43→Aβ40 and Aβ48→Aβ45→Aβ42→Aβ38 (8).

**Figure 1:**
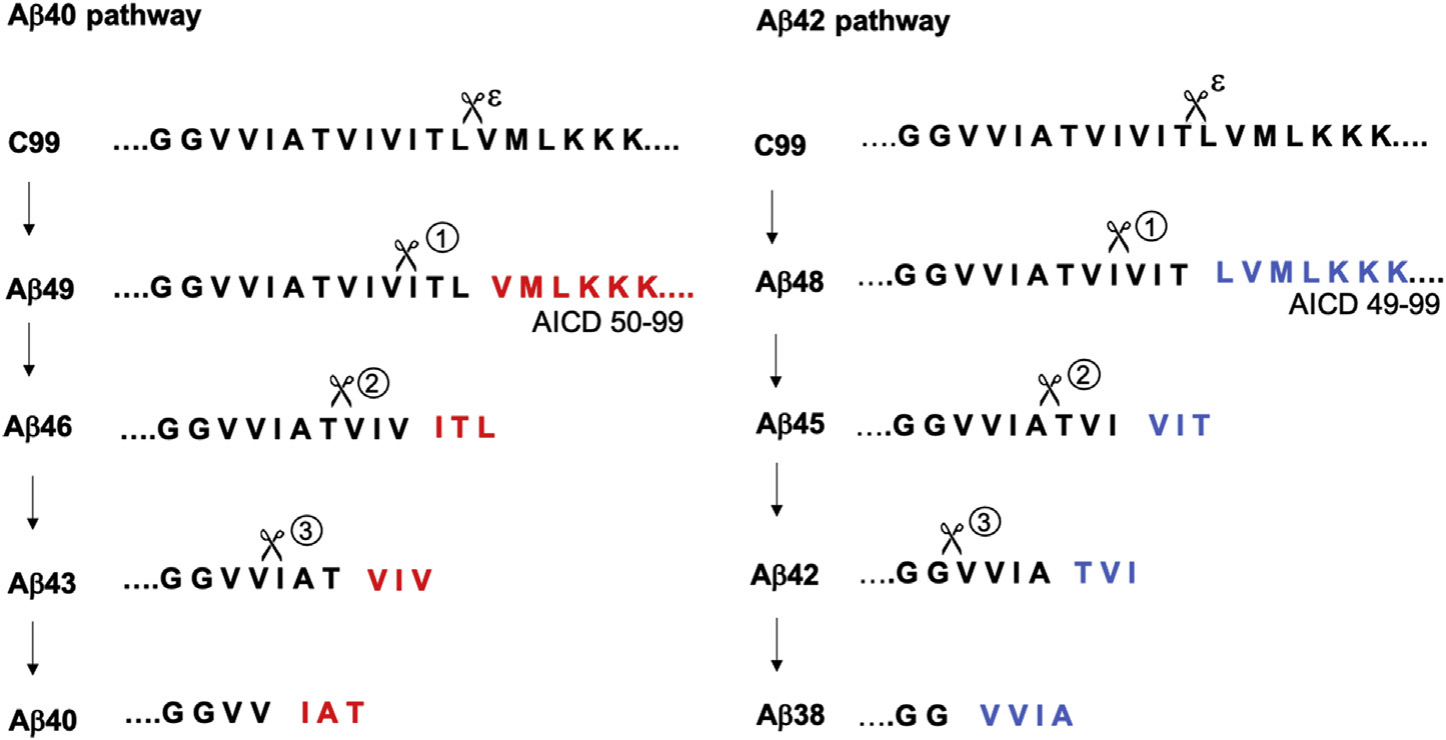
**Successive proteolysis in the TMD of APP substrate C99 by γ-secretase proceeds *via* two pathways resulting in either Aβ40 or Aβ42.** First, ε cleavage takes place at one of two sites to generate Aβ49 and AICD 50 to 99 or Aβ48 and AICD 49 to 99. Aβ49 and Aβ48 are then sequentially cleaved, releasing small tri- and tetrapeptides as shown. Small peptides generated in the Aβ40 pathway are ITL, VIV, and IAT, while those generated in the Aβ42 pathway are VIT, TVI, and VVIA.

Understanding how FAD mutations initiate the disease process requires a comprehensive and quantitative analysis of all the proteolytic steps carried out by γ-secretase on APP. Such analysis is challenging, as Aβ peptides of 45 residues and longer are difficult to detect and quantify by mass spectrometry (MS) and have no specific antibodies for ELISA. We have previously shown that five different FAD-mutant γ-secretase complexes are deficient in carboxypeptidase function (9) and increase the proportion of Aβ that is 45 residues and longer (10). However, this limited study was accomplished using a long, hand-cast urea-PAGE system and western blotting, which is technically challenging, difficult to quantify, and insufficient for separating Aβ46 through Aβ49. Here we employ liquid chromatography coupled to tandem mass spectrometry (LC-MS/MS) as a central method to quantify the tri- and tetrapeptide coproducts to determine the levels of each Aβ peptide produced by purified γ-secretase from wild-type and 14 different FAD-mutant APP substrates. Few of these mutations elevated Aβ42, and not all increased the Aβ42/Aβ40 ratio. In contrast, we find that all 14 FAD mutations altered processive proteolysis by γ-secretase to elevate levels of Aβ peptides of 45 residues and longer. Moreover, we find that long Aβ intermediates are not released and reassociated with the enzyme for subsequent trimming; instead, they remain bound for further processing.

## Results

### Effects of APP FAD mutations on Aβ42/Aβ40

**Figure 2:**
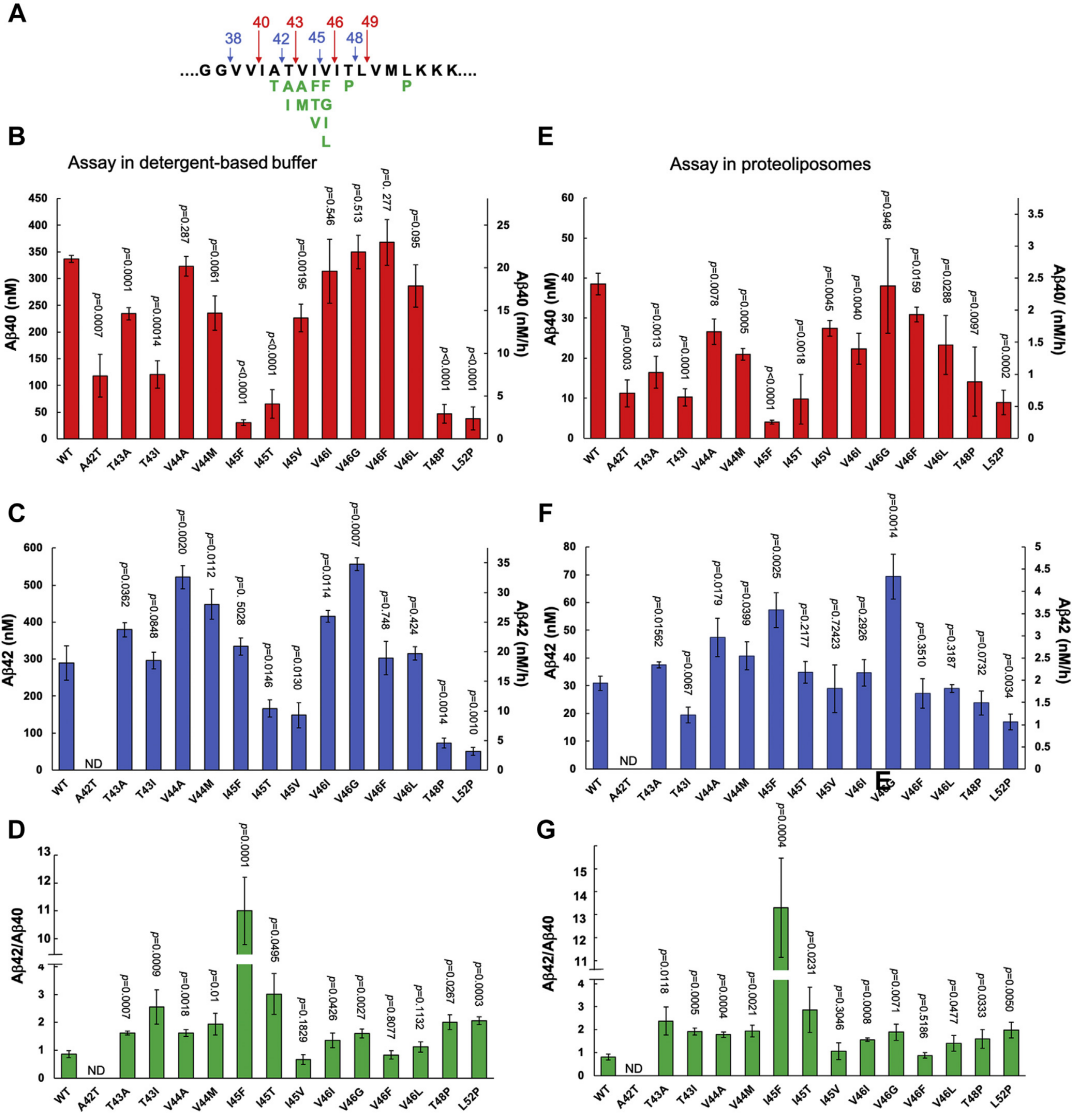
**Effects of APP TMD familial Alzheimer’s disease (FAD) mutations on Aβ40 and Aβ42 production from purified γ-secretase as determined by specific ELISAs.***A*, sequence of the APP TMD showing the two pathways of sequential cleavage by γ-secretase (*arrows*) and missense mutations causing FAD (*green*). *B*–*D*, concentration of (*B*) Aβ40 and (*C*) Aβ42 and (*D*) the ratio of Aβ42 to Aβ40 determined by ELISA from proteolytic processing by γ-secretase of WT and FAD mutants of APP substrate C100-FLAG in the detergent-solubilized enzyme reaction system. *E*–*G*, concentration of (*E*) Aβ40 and (*F*) Aβ42 and (*G*) the ratio of Aβ42 to Aβ40 determined by ELISA from proteolytic processing by γ-secretase of WT and FAD mutants of APP substrate C100-FLAG in the proteoliposome enzyme reaction system. Note that Aβ42 levels cannot be determined by ELISA for the A42T mutation, as this leads to mutation in the C-terminal epitope. *p* values were calculated using Student’s *t*-test. Error bars = S.D., n = 3.

We first investigated the effects of the APP FAD mutations on the production of Aβ42 and Aβ40 peptides and the ratio of Aβ42 to Aβ40 peptides. For substrate, we used C100-FLAG, a standard recombinant C99-based substrate with an N-terminal methionine and a C-terminal FLAG epitope tag. We and others have employed this substrate routinely in biochemical γ-secretase assays, as it recapitulates C99 processing by the protease complex (11, 12). We expressed and purified C100-FLAG along with 14 different FAD-mutant versions (Fig. 2*A*). We also expressed γ-secretase in suspended human embryonic kidney (HEK) 293 cells using a tetracistronic construct encoding all four components (presenilin-1, nicastrin, Aph-1aL, and Pen-2) (13) and then purified the complex to homogeneity. The WT and FAD-mutant substrates were subjected to proteolysis by 30 nM of γ-secretase under saturating conditions (5 μM of substrate) in two different systems, solubilized in detergent or reconstituted in proteoliposomes. These two systems were used in order to establish whether the detergent-solubilized conditions would give results regarding Aβ40 *versus* Aβ42 production that are similar to that of the more native-like conditions of proteoliposomes, as the high lipid concentrations of the latter would interfere with subsequent MS analysis of small peptide products. Levels of Aβ40 and Aβ42 derived from proteolysis of the C100 substrates under both conditions were quantified by specific ELISAs (Fig. 2, *B*, *C*, *E*, and *F*). The detergent-solubilized condition produced approximately tenfold more Aβ40 and Aβ42 peptides. However, the relative effects of the FAD mutations compared with WT substrate on both Aβ40 and Aβ42 are closely similar between the two conditions. Moreover, the ratios of Aβ42 to Aβ40 are nearly identical for all the substrate variants between the two different conditions. The ratios calculated showed that three FAD mutants I45V, V46F, and V46L did not lead to increases in Aβ42/Aβ40 in the detergent-solubilized system. Similarly, I45V and V46F did not exhibit increased Aβ42/Aβ40 in proteoliposomes (V46L reached a statistically significant increase in this system). The similar Aβ42-to-Aβ40 ratios for the detergent-solubilized and proteoliposome systems suggest that proteolytic processing of these C100 substrates by γ-secretase in the solubilized state takes place in a manner similar to what occurs within a lipid bilayer. Note that Aβ42/Aβ40 from WT substrate is higher than what occurs for C99 in cells because these *in vitro* reactions were performed under saturating substrate conditions (14). Nevertheless, relative changes in Aβ42/Aβ40 between WT and FAD-mutant substrates can be determined with confidence. For instance, the I45F mutation gave the highest Aβ42/Aβ40 as expected (12).

### LC-MS/MS analysis of small-peptide coproducts of carboxypeptidase activity with WT and FAD-mutant APP substrates by γ-secretase

The results above suggest that increased Aβ42/Aβ40 is not necessary for the pathogenesis of FAD, as not all FAD mutations in the APP TMD lead to such elevations. Therefore, we sought to analyze and quantify all other possible proteolytic products generated during APP TMD proteolysis by γ-secretase. Quantification of small peptide coproducts (tri- and tetrapeptides) generated during trimming is an indirect way to quantify the production of Aβ46, Aβ43, and Aβ40 along the Aβ40 pathway and the production of Aβ45, Aβ42, and Aβ38 along the Aβ42 pathway. The WT substrate generates ITL, VIV, IAT sequentially in the Aβ40 pathway and VIT, TVI and VVIA sequentially in the Aβ42 pathway (Fig. 1).

**Figure 3:**
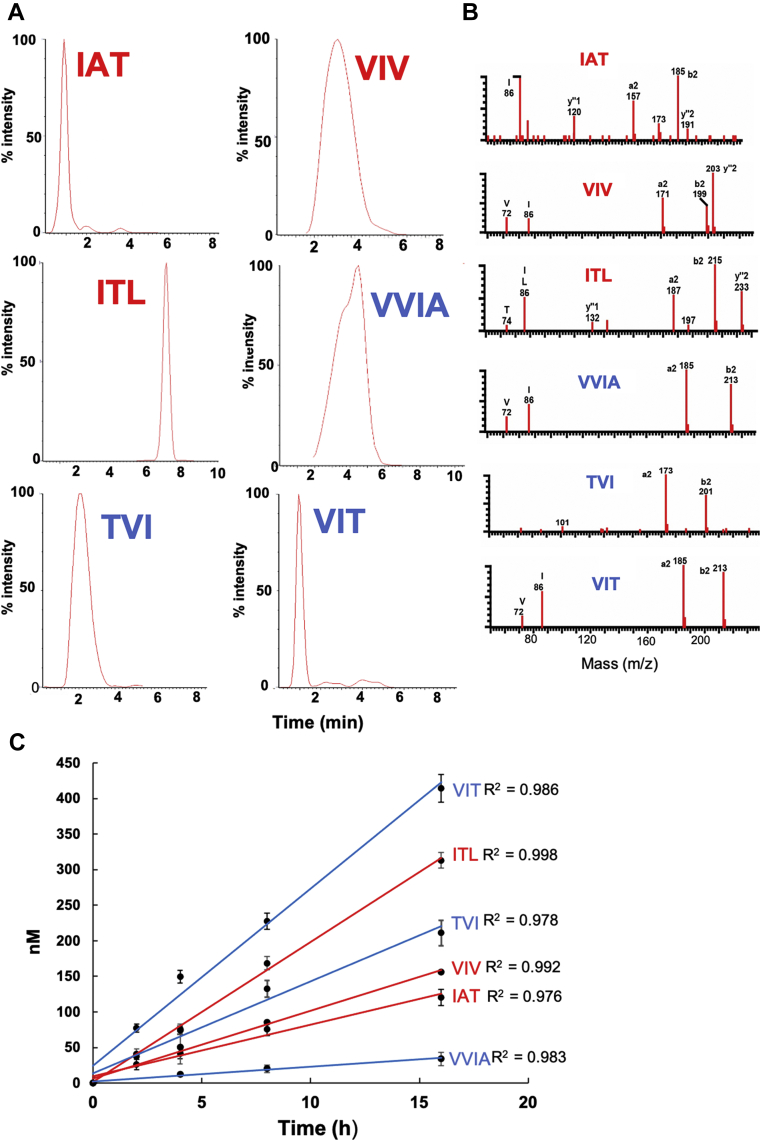
**LC-MS/MS detection and quantification of tri- and tetrapeptides generated during processive proteolysis of WT APP substrate (C100-FLAG) by γ-secretase.***A*, LC-MS/MS of small peptides released after γ-secretase digestion of WT substrate for 16 h. Chromatograms are selected ion plots of the three most abundant sequence-specific product ions, selected with a 0.03 unit window. *B*, MS fragmentation of peptides annotated with sequence-indicative ions for each chromatograms. *C*, time course experiment for the generation of small peptides.

An LC-MS/MS method was developed, based on Takami *et al.* (8), to analyze and quantify the small-peptide coproducts generated from the trimming of WT C100-FLAG substrate by γ-secretase. First, the mixtures from the detergent-based system and from the proteoliposome-based system were subjected to LC-MS/MS analysis to detect small peptides. All small peptides expected to be generated were detected in the detergent-solubilized assay system (Fig. 3, *A* and *B*). However, the same method failed to detect peptides from the proteoliposome system (data not shown), likely due to low concentrations of the products from the proteoliposome system, as evident from the ELISA assays in Fig. 2, as well as interference by the high levels of lipids.

For quantification of small-peptide production, standard curves of each peptide were generated by plotting the concentration of pure synthetic peptide *versus* the integrated areas of the three most abundant ion fragments from MS/MS. The standard curves show excellent linearity from 62.5 nM to 1000 nM for ITL, VIV, IAT, VIT, and TVI peptides, while VVIA showed excellent linearity from 31.25 nM to 1000 nM (Fig. S1). With all standard curves established, each peptide generated in the detergent-solubilized γ-secretase cleavage assay with WT C100-FLAG was monitored and quantified after 2, 4, 8, and 16 h reaction times. All small peptides from WT substrate were generated in a linear fashion with time up to 16 h (Fig. 3*C*). Consistent with the sequence of the carboxypeptidase trimming steps, Aβ40 pathway peptides were generated with relative rates ITL > VIV > IAT, and Aβ42 pathway peptides were generated with relative rates VIT > TVI > VVIA.

**Figure 4:**
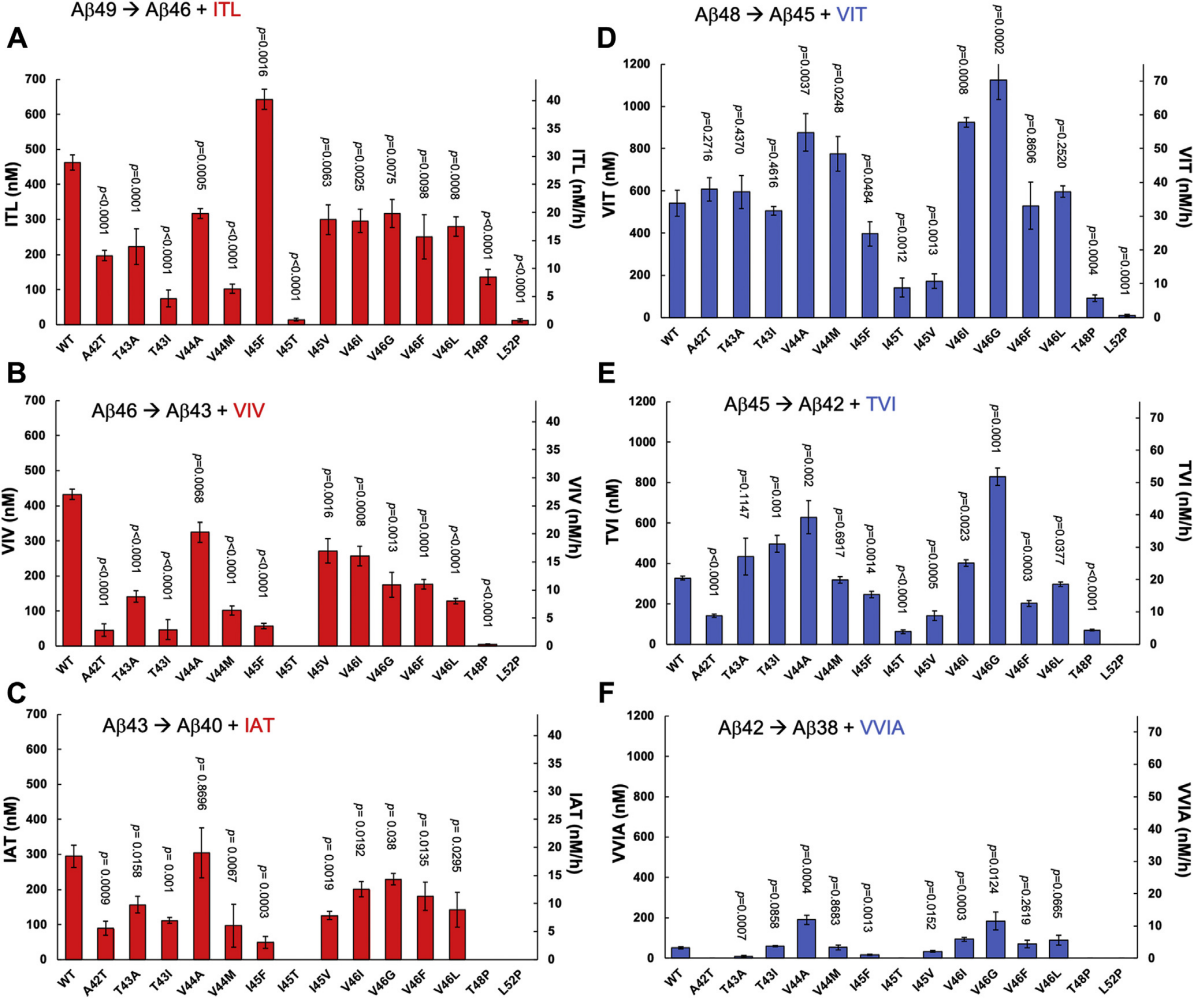
**Comparison of small peptides generated from WT and FAD mutants of APP substrate C100-FLAG by purified γ-secretase.***A*–*C*, quantification of the first, second, and third trimming steps in the Aβ40 pathway with WT and FAD-mutant substrate, measuring the concentration of tripeptides (*A*) ITL (conversion from Aβ49 to Aβ46), (*B*) VIV (conversion from Aβ46 to Aβ43), and (*C*) IAT (conversion from Aβ43 to Aβ40). *D*–*F*, quantification of the first, second, and third trimming steps in the Aβ42 pathway with WT and FAD-mutant substrate, measuring the concentration of tri- or tetrapeptides (*D*) VIT (conversion from Aβ48 to Aβ45), (*E*) TVI (conversion from Aβ45 to Aβ42), and (*C*) VVIA (conversion from Aβ42 to Aβ38). *p* values were calculated using Student’s *t*-test., error bars = S.D., n = 3.

Next, we quantified small peptides generated by γ-secretase from all 14 FAD-mutant substrates. Thirteen of the 14 mutant substrates produce two unique small peptides each—one from the Aβ40 pathway and one from the Aβ42 pathway—that are different from the small peptides generated from trimming of the WT substrate (Table S1), due to mutation within the trimmed Aβ sequence. Only the L52P-mutant substrate is processed to the same set of small peptides generated from the WT substrate. Standard curves for all the possible small-peptide coproducts from processive proteolysis of all 14 FAD-mutant substrates were generated and showed excellent linearity from 62.5 nM to 1000 nM (Fig. S1). Using these standard curves, each small peptide generated during proteolytic trimming for WT and all 14 FAD-mutant substrates was quantified (Fig. 4).

For the Aβ40 pathway, levels of ITL produced from the Aβ49→Aβ46 trimming step (measuring the production of Aβ46 and degradation of Aβ49) decreased with all of the FAD-mutant substrates except for I45F when compared with WT substrate (Fig. 4*A*). Levels of downstream trimming products VIV from Aβ46→Aβ43 (a measure of production of Aβ43 and degradation of Aβ46) and IAT from Aβ43→Aβ40 (measuring production of Aβ40 and degradation of Aβ43) also decreased except for V44A, for which there were no significant changes compared with WT (Fig. 4, *B* and *C*). For the Aβ42 pathway, levels of VIT produced from the Aβ48→Aβ45 trimming step (production of Aβ45 and degradation of Aβ48) were significantly higher compared with WT substrate with mutant substrates V44A, V44M, V46I, and V46G and significantly lower with mutants I45F, I45T, I45V, T48P, and L52P (Fig. 4*D*). Levels of the next trimming product TVI, from Aβ45→ Aβ42 (production of Aβ42 and degradation of Aβ45), were increased in T43I, V44A, V46I, and V46G compared with WT and decreased in A42T, I45F, I45T, I45V, V46F, V46L, T48P, and L52P (Fig. 4*E*). Levels of the final trimming product VVIA, from Aβ42→Aβ38 (production of Aβ38 and degradation of Aβ42), also increased from substrate mutants V44A, V46I, V46G, and V46L compared with WT and decreased from A42T, T43A, I45F, I45T, I45V, T48P, and L52P mutants. (Fig. 4*F*).

### Analysis of AICD products from ε cleavage of APP WT and FAD-mutant substrates by γ-secretase

**Figure 5:**
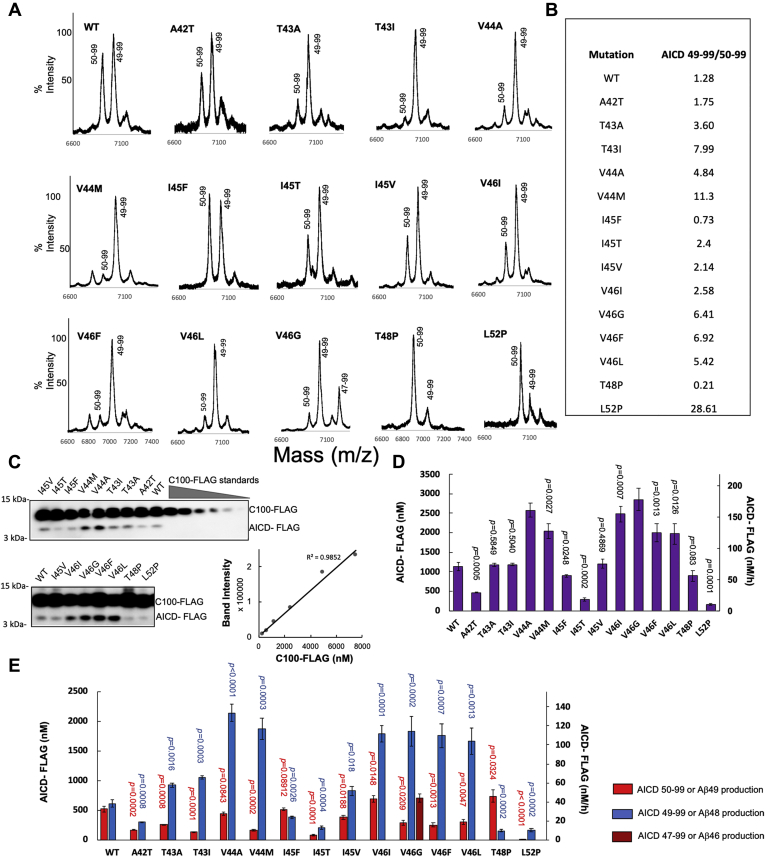
**Quantification of ε cleavage products AICD 50 to 99 and AICD 49 to 99 produced from WT and FAD mutants of APP substrate C100-FLAG by purified γ-secretase, as an indirect measure of Aβ48 and Aβ49 generation.***A*, MALDI-TOF MS detection of AICD 50 to 99 and AICD 49 to 99 products. *B*, ratios of peak intensities of AICD 49 to 99 to AICD 50 to 99 determined by MALDI-TOF MS. *C*, anti-FLAG immunoblot of total AICD-FLAG levels. *D*, quantification by densitometry of total AICD-FLAG levels from immunoblot. Purified C100-FLAG at a range of known concentrations was used to generate a standard curve. *E*, quantification of both AICD 49 to 99 and AICD 50 to 99 using total AICD levels determined from immunoblot and intensity ratios determined from MALDI-TOF MS. Level of AICD 47 to 99 produced from the V46G mutant substrate was also determined. *p* values were calculated using Student’s *t*-test. Error bars = S.D., n = 3.

Up to this point, production and degradation of all Aβ peptides were quantified indirectly by analyzing coproducts (small peptides) by LC-MS/MS, except for production of Aβ48 and Aβ49. To quantify Aβ48 and Aβ49 generated by ε cleavage of APP substrate, levels of coproducts AICD 49 to 99 and AICD 50 to 99 peptides were determined using a combination of matrix-assisted laser desorption/ionization time-of-flight (MALDI-TOF) MS and quantitative western blotting. First, AICD species generated in the detergent-solubilized system were immunoprecipitated with anti-FLAG antibodies and monitored by MALDI-TOF MS (Fig. 5*A*). The ratios of signal intensities corresponding to AICD 49 to 99 to AICD 50 to 99 were calculated and tabulated for enzyme reactions with WT and all mutant substrates (Fig. 5*B*). The ratio of AICD 49 to 99 to AICD 50 to 99 from WT C100-Flag substrate was 1.28, and this ratio is increased with all the mutant substrates except for I45F and T48P, for which it is decreased. This indicates that most of the FAD mutations shift ε cleavage in favor of Aβ48 compared with WT substrate, consistent with previous reports (15, 16). In contrast, the I45F and T48P mutations favor ε cleavage producing Aβ49 (12). Unique among the substrate variants, the V46G mutation led to ε cleavage at three sites, forming AICD 47 to 99 along with AICD 49 to 99 and AICD 50 to 99. MALDI-TOF MS analysis of AICD products generated from the proteoliposome system and immunoprecipitated with anti-FLAG antibodies gave closely similar results (Fig. S2), again demonstrating that the detergent-solubilized system recapitulates γ-secretase cleavage of APP substrate as it occurs within a lipid bilayer.

Because quantification of AICD 49 to 99 and AICD 50 to 99 levels by MALDI-TOF MS as above would require standards of these specific AICD isoforms that we did not possess, the same reaction mixtures were subjected to quantitative western blotting using anti-FLAG primary antibodies (Fig. 5*C*). Known concentrations of C100-FLAG were run in parallel to make a calibration curve, plotting band intensity with concentration of FLAG-tagged protein (Fig. 5*C*). From this standard curve, the concentration of total AICD-FLAG product generated in the enzyme reaction mixtures can be quantified (Fig. 5*D*). AICD product generated by γ-secretase from WT substrate was 1.06 ± 0.19 μM, giving a k_cat_ of 2.2 ± 0.38 h^−1^, consistent with previously reported values (17, 18) and providing further illustration of the remarkably slow rate of reaction by intramembrane proteases (19). Quantification of the total AICD product levels for each mutant substrate revealed increased ε cleavage compared with WT substrate for mutations V44A, V44M, V46I, V46G, V46F, and V46L and decreased ε cleavage for mutations A42T, I45F, I45T, and L52P mutant substrates. Mutations T43A, T43I, I45V, and T48P showed no change in total AICD production compared with that seen with WT substrate. The concentration of each AICD product (AICD 49–99 and AICD 50–99) could then be calculated using the total AICD level determined by quantitative western blot and the ratio of AICD 49 to 99 to AICD 50 to 99 determined from MALDI-TOF MS (Fig. 5*E*). The calculated concentrations of AICD 49 to 99 and AICD 50 to 99 thereby provide the level of production of coproducts Aβ48 and Aβ49, respectively. In the case of V46G, detection of coproduct AICD 47 to 99 allowed determination of the level of Aβ46 produced directly through ε cleavage of this mutant substrate.

### Calculation of net Aβ species for WT and FAD-mutant APP substrates

**Figure 6:**
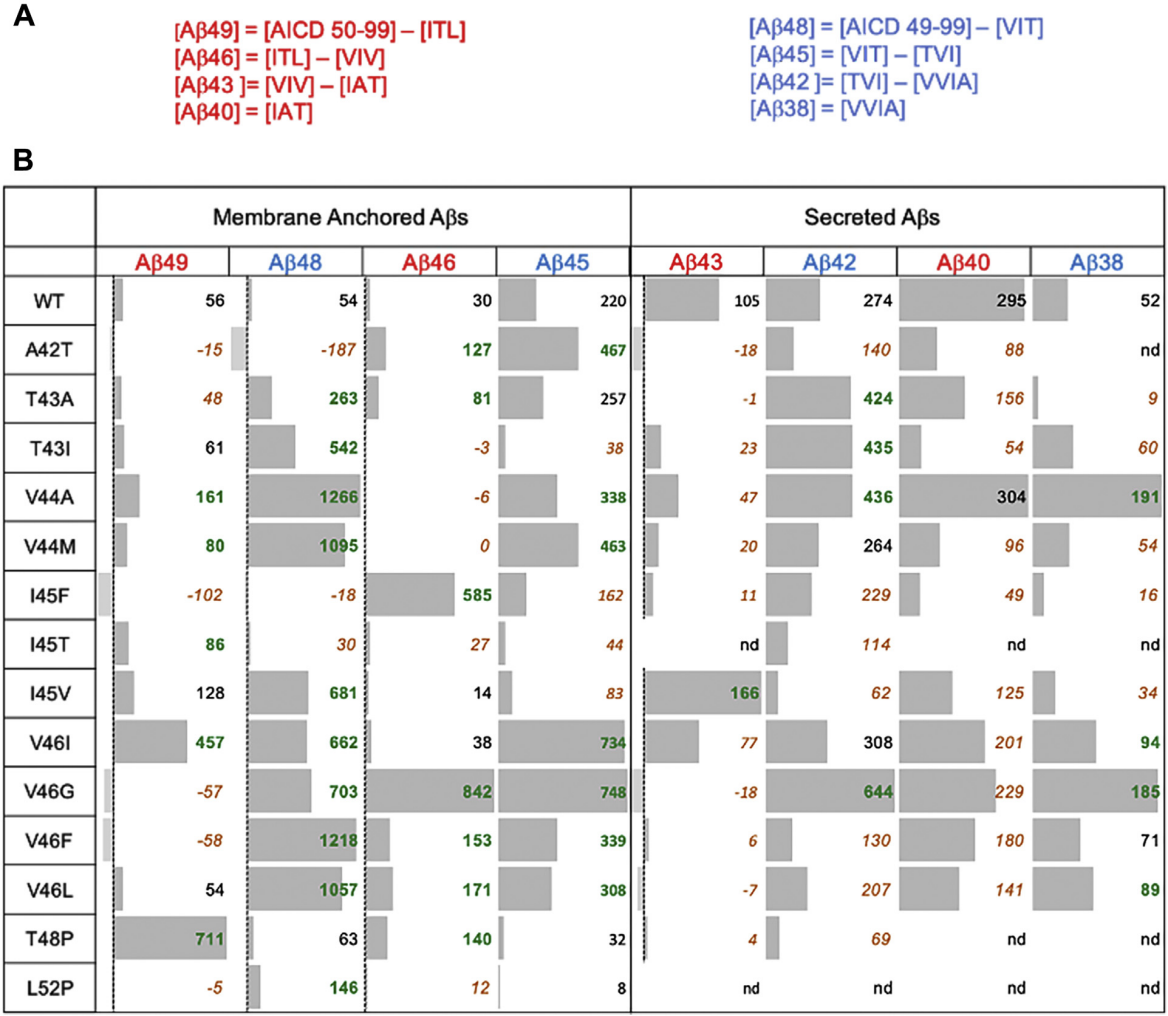
**Net levels of Aβ peptides generated by γ-secretase from WT and FAD-mutant APP substrates.***A*, net concentration of each Aβ was calculated by subtracting the concentration of small peptide coproduct of degradation from the concentration of peptide coproduct of production. Note that each FAD mutation results in mutant forms of certain products. *B*, net concentration (nM) of Aβ peptides generated by 30 nM γ-secretase from a saturating level (5 μM) of WT or FAD-mutant APP substrate. Significant increases in a specific Aβ peptide from FAD-mutant substrate compared with WT substrate are denoted in green bold, and decreases are denoted in brown italics. Note that for V46G, 710 nM of Aβ46 generated through direct ε cleavage was added to the calculation.

The net level of each species of Aβ generated by γ-secretase from each APP substrate was calculated from the quantified coproducts (small peptides and AICD species). Net levels of Aβ peptides were determined by subtracting the concentration of small peptide coproduct of degradation from the concentration of peptide coproduct of production (Fig. 6*A*). The net concentration of each Aβ peptide species for WT and mutant substrates are illustrated and tabulated in Figure 6*B*, with net increases compared with WT substrate in green bold and net decreased in red italics. For WT substrate, net Aβ40 and Aβ42 levels determined by this indirect method are closely similar to that determined from the same enzyme reaction by specific ELISAs (Aβ40: 295 ± 31 nM by LC-MS/MS, 337 ± 5 nM by ELISA; Aβ42: 274 ± 15 nM by LC-MS/MS, 281 ± 33 nM by ELISA). This provides strong validation of the LC-MS/MS method for determining the net concentrations of each Aβ peptide.

γ-Secretase processing of all FAD mutations in the APP TMD led to increased net levels in one or more Aβ peptides of 45 to 49 residues in length. The A42T mutation led to the elevation of Aβ46 and Aβ45 levels. The T43A mutation elevated Aβ48 and Aβ46 levels, while the T43I mutation increased Aβ48. Both V44A and V44M mutations led to increased Aβ49, Aβ48, and Aβ45 species. For the I45F mutant, Aβ46 peptide is elevated, consistent with our previous study showing that aromatic amino acids such as phenylalanine are not tolerated in the P2’ position (12): The I45F mutation places phenylalanine in the P2’ position for the Aβ46→Aβ43 trimming step, thereby blocking this proteolytic event. I45T and I45V mutations increased Aβ49 and Aβ48, respectively. The V46I mutant elevated Aβ49, Aβ48, and Aβ45, while V46G, V46F, and V46L resulted in increased Aβ48, Aβ46, and Aβ45 levels. The T48P mutant elevated Aβ49 and Aβ46, and L52P increased Aβ48. Only four mutations elevated Aβ42 (T43A, T43I, V44A, and V46G), and one (I45V) elevated the amyloidogenic Aβ43 peptide (20, 21, 22) compared with WT substrate. As seen by ELISA, mutations I45V and V46F did not increase Aβ42/Aβ40. Elevations of these long Aβ peptides from each of the 14 FAD-mutant APP substrates by γ-secretase are due to lower efficiency in the first and/or second carboxypeptidase trimming steps, defined as the percent of Aβ peptide produced that is carried through the next processing step (Fig. S3).

## Discussion

Aβ42 has been long accepted to be the pathogenic Aβ variant in Alzheimer’s disease. This particular Aβ variant is the major component of the cerebral extraneuronal amyloid plaques that characterize the disease, and FAD mutations in APP and the presenilins can elevate the ratio of Aβ42 to Aβ40. However, unambiguous identification of pathogenic assemblies of aggregation-prone Aβ42 and elucidation of neurotoxic mechanisms have been elusive. The recent finding that many presenilin-1 FAD mutations do not elevate Aβ42/Aβ40 (6) raises further concerns about Aβ42 as an essential initiator of pathogenesis. Understanding how FAD mutations alter APP processing by γ-secretase requires a rigorous, comprehensive, and quantitative analysis of all proteolytic steps, but to date this has not been performed for any FAD mutations.

Here we conducted such a study of 14 different FAD missense mutations in the APP TMD and found, surprisingly, that all these mutations led to elevated levels of Aβ peptides of 45- to 49-residues in length. Such elevations are due to deficient first and/or second trimming steps by γ-secretase. The direct measure of these long Aβ peptides is challenging, as there are no specific antibodies for any of them and they are difficult to detect and quantify by MS. We previously reported in a limited study that five FAD-mutant presenilin-1/γ-secretase complexes were deficient in carboxypeptidase activity (9) and increased the proportion of long Aβ peptides (10). However, detecting Aβ45 through Aβ49 required long, hand-cast urea-PAGE systems and western blotting that were difficult to quantify and did not separate Aβ peptides of 46 residues and longer. Moreover, many FAD mutations in the APP TMD lead to mutant forms of these long Aβ peptides that would run aberrantly on gels (23). The ability to quantify the small peptide coproducts by LC-MS/MS, a method originally developed by the Ihara lab (8), provides an indirect means of quantifying each Aβ peptide, by subtracting the level of small peptide coproduct of degradation from the level of small peptide coproduct of formation.

Using WT APP substrate and γ-secretase in a detergent-solubilized assay, we found that levels of Aβ40 and Aβ42 determined by the indirect LC-MS/MS method were closely similar to levels determined by specific ELISAs. These results suggest that Aβ40 and Aβ42 are produced by γ-secretase almost exclusively along the canonical pathways of Aβ49→Aβ46→Aβ43→Aβ40 and Aβ48→Aβ45→Aβ42→Aβ38 and provide confidence that the quantification of other Aβ peptides is likewise accurate. Despite concern that production of Aβ49 and Aβ48 was measured indirectly by a different method (quantification of AICD by western blot in conjunction with MS determination of the ratio of AICD isoforms), the sum of Aβ peptide levels is virtually identical to the total AICD level, as expected (24). Moreover, the sum of Aβ peptides along either the Aβ49→Aβ40 or Aβ48→Aβ38 pathway is equal to the level of the corresponding AICD isoform (Table 1). This internal consistency gives high confidence in the accuracy of the results.

**Table 1:** Summation of Aβ levels from LC-MS/MS (total, Aβ49→Aβ40, and Aβ48→Aβ38) compared with levels of AICD (total, 50–99, and 49–99) quantified by immunoblot and MALDI-TOF MS

Mutation	Total aβs	Total AICD	Aβ40+Aβ43+Aβ46+Aβ49	AICD 50–99	Aβ38+Aβ42+Aβ45+Aβ48	AICD 49–99
WT	1089	1062	486	465	602	596
A42T	823	468	215	169	608	298
T43A	1241	1096	285	238	955	858
T43I	1217	1179	139	132	1077	1047
V44A	2746	2584	513	441	2232	2142
V44M	2075	2035	196	164	1878	1871
I45F	1054	891	646	513	408	378
I45T	303	287	114	84	189	203
I45V	1297	1206	435	384	862	822
V46I	2574	2477	774	690	1800	1787
V46G	3351	2856	361^a^	285	2281	1827
V46F	2100	2001	340	252	1759	1748
V46L	2029	1958	367	304	1662	1653
T48P	1022	895	857	738	165	155
L52P	167	162	12	5	155	156

a 710 nM Aβ46 produced directly through ε proteolysis of the V46G mutant substrate was subtracted, as this portion of Aβ46 was not produced through ε proteolysis that generates coproduct AICD 50 to 99.

For comprehensive analysis of γ-secretase processing of 14 different FAD-mutant APP substrates compared with WT substrate, we continued to use the detergent-solubilized system, as this provided almost tenfold more Aβ40 and Aβ42 than the proteoliposome system. Moreover, the detergent-solubilized system avoided the problem presented by the proteoliposome system of high levels of lipids interfering with small peptide separation and detection. Relative effects of the 14 FAD-mutant substrates on Aβ40 and Aβ42 levels compared with WT substrate in the two systems were virtually identical, as were effects on the ratio of AICD 50 to 99 to AICD 49 to 99, demonstrating that the detergent-solubilized system gives results that are reflective of what occurs within a lipid bilayer. In both systems, most but not all of the 14 FAD-mutant substrates elevated Aβ42/Aβ40, similar to findings from a recent analysis of 138 FAD-mutant PSEN1/γ-secretase complexes (6). Thus, inconsistency with the hypothesis that increased Aβ42/Aβ40 is necessary for pathogenicity in FAD is extended to APP mutations.

Quantification of all the small peptide products from γ-secretase processing of the 14 FAD-mutant substrates by LC-MS/MS required generating standard curves for each small peptide, including mutant versions of each (32 small peptide standards in all). In this way, the degree of each carboxypeptidase trimming step could be quantified. Results from quantitative western blotting of total AICD production and MS determination of the ratio of the two AICD isoforms allowed quantitative determination of the production of Aβ48 and Aβ49 from each mutant substrate as well. With all these data in hand, knowing the extent of individual Aβ production and degradation, levels of each of the eight different Aβ peptides generated from γ-secretase processing of each of the 14 FAD-mutant APP substrates could be determined and compared with that observed for WT substrate. The effects of FAD mutations on Aβ40 and Aβ42 and Aβ42/Aβ40 were generally similar to those determined by ELISA. Only five of the 14 mutations elevated Aβ42 and two of the mutations (I45V and V46F) did not increase Aβ42/Aβ40.

As with WT substrate, for almost every mutant substrate, the sum of Aβ peptides was nearly equal to the total AICD level, and the sum of Aβ peptides along the Aβ49→Aβ40 or Aβ48→Aβ38 pathway was closely similar to levels of the respective AICD isoforms (Table 1). The two clear exceptions were the sum of Aβ peptides along the Aβ48→Aβ38 pathway from A42T substrate and the sum of Aβ peptides along the Aβ49→Aβ40 pathway from V46G substrate. In the case of A42T, the mutation introduces the possibility of a noncanonical tripeptide trimming of Aβ42→Aβ39 with coproduct VIT, the same tripeptide coproduct formed by the trimming of Aβ48→Aβ45. This may explain the large negative value calculated for the Aβ48 level with this mutant substrate: more VIT is produced than is possible from the level of Aβ48 production. The discrepancy in the Aβ49→Aβ40 pathway with the V46G mutant substrate is apparently because Aβ46 was primarily produced directly by ε cleavage and not trimmed efficiently. Once the directly produced Aβ46 is subtracted, the sum of the levels of Aβ peptides in the Aβ49→Aβ40 pathway is similar to the determined level of AICD 50 to 99. That all the other pathway levels match the corresponding AICD isoforms provides validation that the level of each Aβ species is calculated accurately. This is remarkable considering that all the mutant substrates except L52P introduce mutations into multiple Aβ species.

The results with the L52P substrate mutation provide further insight into the nature of processive proteolysis. This mutant substrate is cleaved to Aβ48 almost exclusively. Even though Aβ48 is the wild-type sequence, very little is trimmed to Aβ45. In contrast, Aβ48 produced from WT substrate is effectively trimmed. This indicates that the Aβ intermediates do not dissociate from the enzyme and then reassociate for trimming, as in such a scenario the trimming of Aβ48 would be the same whether this peptide was generated from WT or L52P-mutant substrate. Two mechanisms for the reduced Aβ48→Aβ45 trimming from the L52P substrate seem possible. One is that the L52P-mutant AICD product does not dissociate from the enzyme as readily as WT AICD. Dissociation of AICD should be necessary to allow the next processing step. The second possibility is that the interaction of L52P-mutant substrate with γ-secretase and subsequent ε cleavage to Aβ48 leaves the enzyme in a different conformation that has a higher energy of activation for carrying out the next processing step. Interestingly, the processing of Aβ46→Aβ43 from the T48P-mutant substrate is likewise nearly abolished, despite the fact that Aβ46 produced from this mutant is the wild-type sequence. These observations present opportunities to further explore mechanisms by which FAD mutations affect specific trimming steps to elevate long Aβ peptides.

**Figure 7:**
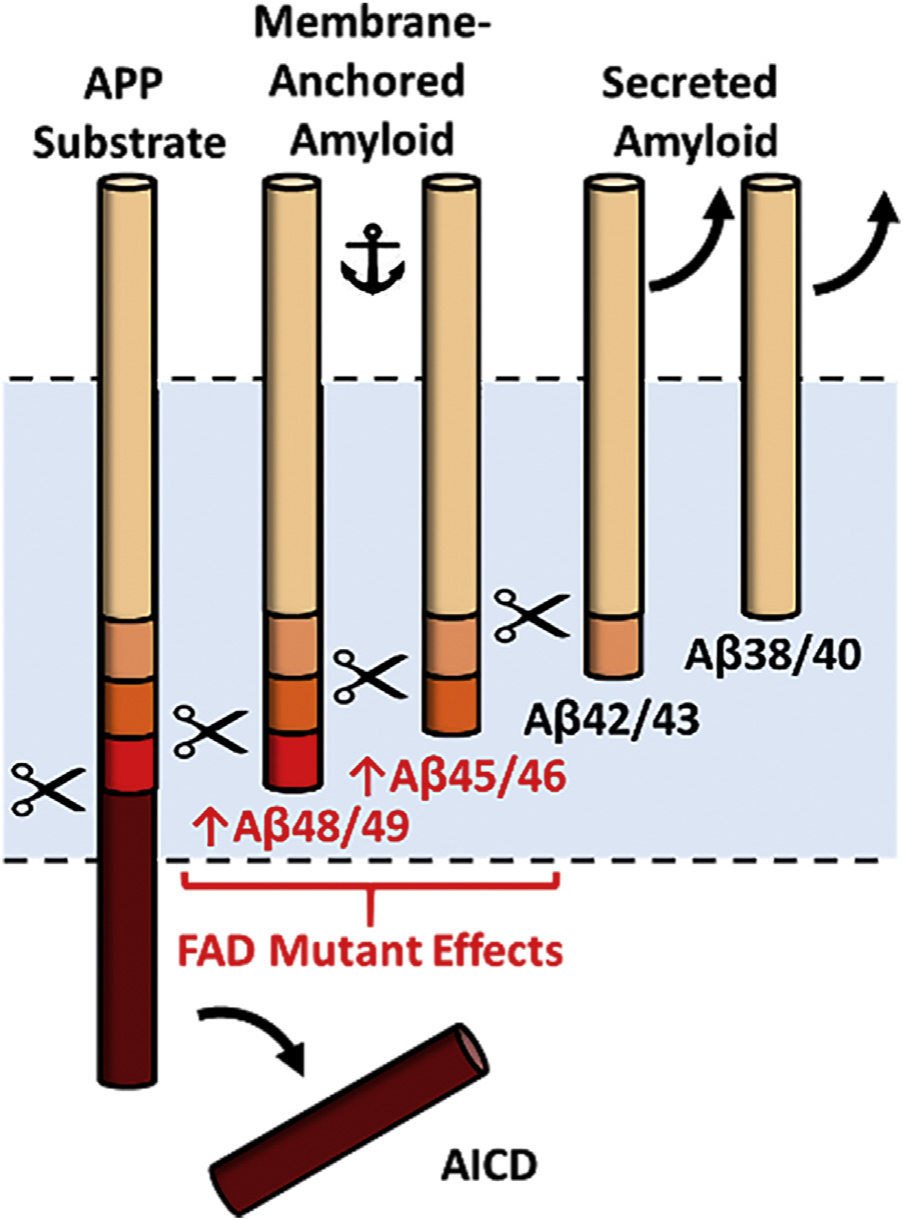
**FAD-mutant APP substrates increase levels of long membrane-anchored Aβ peptides**.

Most remarkable is the finding that each of the 14 different FAD mutations in the APP TMD leads to less efficient trimming of Aβ peptides ranging from 45 to 49 residues in length. These peptides contain most of the APP TMD and are membrane-anchored and not secreted (25) (Fig. 7). Nothing is known about any possible physiological or pathological roles for the long, membrane-anchored Aβ peptides, and they have been largely considered only as intermediates to the shorter secreted forms. Notwithstanding, a possible role of these peptides in the pathogenesis of AD was raised by Ihara and colleagues in their seminal report on the discovery of these intermediates (25). Our collective findings here consistently point to these enigmatic long Aβ peptides as potentially pathogenic, at least in FAD. How these peptides might affect FAD pathology or progression can only be speculated presently but could conceivably involve their assembly into oligomers and membrane pore formation or lateral diffusion and interaction with other membrane proteins. In any case, the inability of γ-secretase to effectively process Aβ peptides of 45 residues and longer appears to be intimately linked to the pathogenesis of FAD.

## Experimental procedures

### Expression and purification of C100-FLAG substrates

All FAD mutations in the C100-FLAG construct in the pET22b vector (11) were introduced by site-directed mutagenesis (QuikChange Lightning Site Directed Mutagenesis kit, Agilent). The wild-type and FAD-mutant constructs were then transformed into *E. coli* BL21 cells. *E. coli* BL21 cells were grown in LB media at 37 °C with continuous shaking to OD_600_ 0.6, whereupon cells were induced with 0.5 mM IPTG and further grown for 4 h. Cells were pelleted, resuspended in lysis buffer consisting of 10 mM Tris pH 8, 1% Triton X-100, and 150 mM NaCl, and lysed by French press. The resulting lysate was centrifuged and incubated with anti-FLAG M2-agarose beads (Sigma-Aldrich) for 16 h at 4 °C. The beads were then washed 4× with lysis buffer, and protein eluted with 100 mM glycine, pH 2.7 with 0.25% NP-40 and neutralized with Tris buffer. Purity of each C100-FLAG substrate was analyzed by SDS/PAGE with Coomassie staining and by western blotting with anti-FLAG antibodies.

### γ-Secretase assays

γ-Secretase expression was carried out using a pMLINK tetracystronic vector encoding the four components of the protease complex (12, 13). Purification and assays were carried out as previously described (26, 27, 28). Briefly, for the detergent-solubilized assay, 30 nM γ-secretase was preincubated for 30 min at 37 °C in assay buffer composed of 50 mM HEPES pH 7.0, 150 mM NaCl, and 0.25% 3-[(3-cholamidopropyl)dimethylammonio]-2-hydroxy-1-propanesulfonate (CHAPSO) supplemented with 0.1% phosphatidylcholine and 0.025% phosphatidylethanolamine. Reactions were initiated by addition of purified C100-FLAG substrate (final concentration 5 μM) and incubation at 37 °C for various times. The reactions were stopped by flash freezing in liquid nitrogen and stored at −20 °C until analysis. For the proteoliposome assay, 30 nM purified γ-secretase was dissolved into total brain lipid extract (Avanti) in 50 mM HEPES pH 7.0, 150 mM NaCl, 0.25% CHAPSO as described (28). The mixture was incubated with SM-2 biobeads (Bio-Rad) for 2 h at 4 °C for the removal of detergent. Biobeads then were removed, and the resulting proteoliposome solution was mixed with 5 μM final concentration of recombinant C100-FLAG substrates. The reaction was initiated by incubation at 37 °C and carried out for 16 h. The proteolytic products from enzyme reaction mixtures from both methods were analyzed by ELISA, immunoblot, and mass spectrometry as described below.

### Mass spectrometric detection of AICD products

After 16 h incubation of γ-secretase reaction mixtures, FLAG-tagged peptides were immunoprecipitated with anti-FLAG M2 beads (SIGMA) in immunoprecipitation buffer (IP) consiting10 mM 2-(N-morpholino) ethanesulfonic acid (MES) pH 6.5, 10 mM NaCl, 0.05% n-dodecyl-β-D-maltoside (DDM) detergent for 16 h at 4 °C. The beads were washed with DDM free IP buffer two times. FLAG-tagged peptides were then eluted from the anti-FLAG beads with acetonitrile: water (1:1) with 0.1% trifluoroacetic acid. AICD-FLAG proteolytic products were detected using a Bruker UltraFlex III MALDI-TOF mass spectrometer.

### Immunoblotting of AICD products

Samples from γ-secretase reaction mixtures and C100-FLAG standards were subjected to SDS-PAGE on 4 to 12% bis-tris gels and transferred to PVDF membranes. Membranes were then successively blocked with 5% dry milk for 1 h at ambient temperature, treated with anti-Flag M2 antibodies for 16 h at 4 °C, then washed and incubated with anti-mouse secondary antibodies for 1 h at ambient temperature. Membranes were washed and imaged for chemiluminescence, and bands were analyzed by densitometry.

### Mass spectrometric analysis of tri- and tetra peptide products

Small peptides were analyzed using an ESI Quadrupole Time-of-Flight (Q-TOF) mass spectrometer (Q-TOF Premier, Waters) by LC-MS/MS experiment. 10 ml of the assay mixture and various concentrations of the synthetic peptides (>98% purity, New England Peptide) were dissolved in assay buffer, loaded onto a C18 analytical chromatography column, and eluted with a step gradient of 0.08% aqueous formic acid (A), acetonitrile (B), isopropanol (c), and a 1:1 acetone/dioxane mixture (D). The three most abundant fragments from collision-induced dissociation were identified by tandem MS for each small peptide. To obtain a peptide chromatographic area, the signals from the three most abundant ions were summed using an ion mass width of 0.02 unit. Data were acquired in “V” mode.

## Data availability

All data are included in the article and supporting information.

## Conflict of interest

The authors declare that they have no conflicts of interest with the contents of this article.
