# Supplementary material for: Familial Alzheimer’s disease mutations in amyloid protein precursor alter proteolysis by γ-secretase to increase amyloid β-peptides of ≥45 residues
Source: J Biol Chem. 2021 Jan 12;296:100281. doi: 10.1016/j.jbc.2021.100281 (PMC7948801; doi:10.1016/j.jbc.2021.100281)
Supplement: Supplemental Figures S1–S3 and Table S1 [file mmc1.pdf]

Supplementary Information for

Familial Alzheimer’s disease mutations in amyloid protein precursor alter proteolysis by γ-secretase to elevate amyloid β-peptides of $\geq$45 residues

Sujan Devkota[1], Todd D. Williams[2], and Michael S. Wolfe[1*]

Michael S. Wolfe
Email: mswolfe@ku.edu

This PDF file includes:

Table S1
Figures S1-S3

Table S1. Small peptides generated for WT and FAD mutants during proteolysis. Each mutation except for L52P generates two different peptides, shown in green, that are different from the peptides generated from the WT.

	Aβ40 pathway ←			Aβ42 pathway ←		
**WT**	IAT	VIV	ITL	VVIA	TVI	VIT
**A42T**	ITT	VIV	ITL	VVIT	TVI	VIT
**T43A**	IAA	VIV	ITL	VVIA	AVI	VIT
**T43I**	IAI	VIV	ITL	VVIA	IVI	VIT
**V44A**	IAT	AIV	ITL	VVIA	TAI	VIT
**V44M**	IAT	MIV	ITL	VVIA	TMI	VIT
**I45F**	IAT	VFV	ITL	VVIA	TVF	VIT
**I45T**	IAT	VTV	ITL	VVIA	TVT	VIT
**I45V**	IAT	VVV	ITL	VVIA	TVV	VIT
**V46I**	IAT	VII	ITL	VVIA	TVI	IIT
**V46G**	IAT	VIG	ITL	VVIA	TVI	GIT
**V46F**	IAT	VIF	ITL	VVIA	TVI	FIT
**V46L**	IAT	VIL	ITL	VVIA	TVI	LIT
**T48P**	IAT	VIV	IPL	VVIA	TVI	VIP
**L52P**	IAT	VIV	ITL	VVIA	TVI	VIT

Figure S1

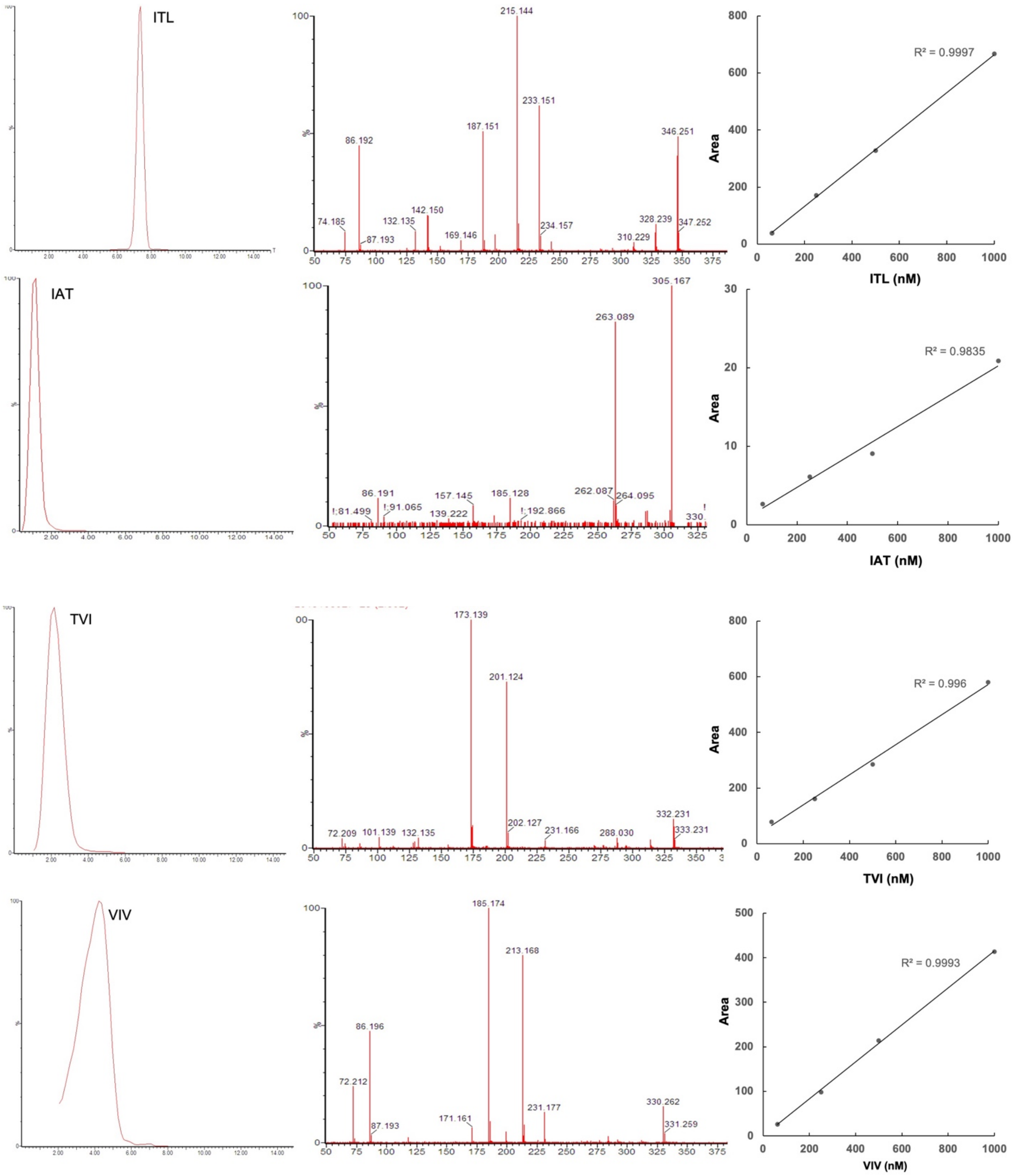

ITL
215.144
233.151
187.151
86.192
346.251
74.185
132.135
142.150
87.193
169.146
234.157
310.229
328.239
347.252
R² = 0.9997
Area
ITL (nM)
IAT
305.167
263.089
86.191
!:81.499
!:91.065
157.145
139.222
185.128
!:192.866
262.087
264.095
330.
R² = 0.9835
Area
IAT (nM)
TVI
173.139
201.124
72.209
101.139
132.135
202.127
231.166
288.030
332.231
333.231
R² = 0.996
Area
TVI (nM)
VIV
185.174
213.168
86.196
72.212
87.193
171.161
231.177
330.262
331.259
R² = 0.9993
Area
VIV (nM)

Figure S1, cont'd

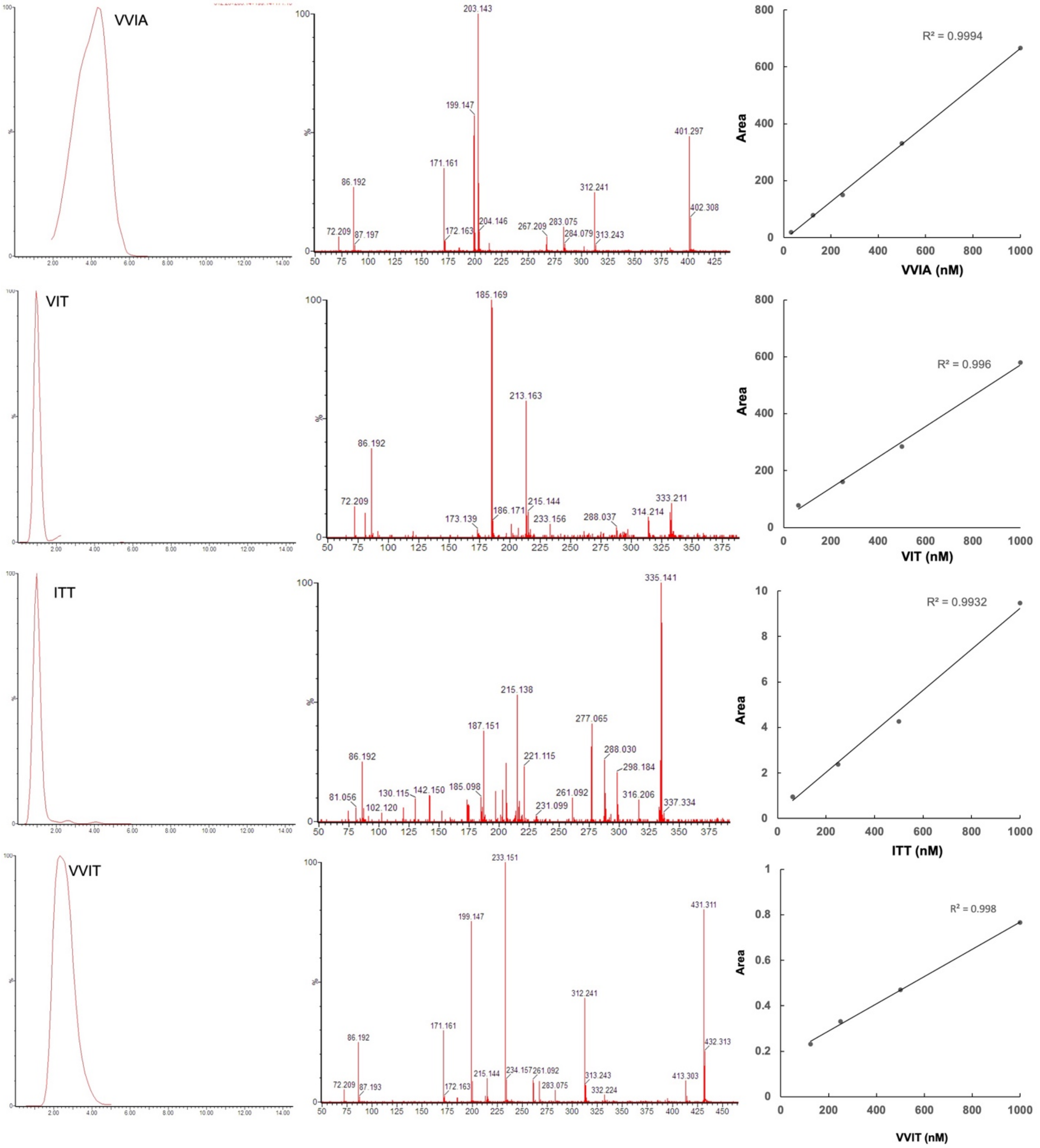

Figure S1, cont'd

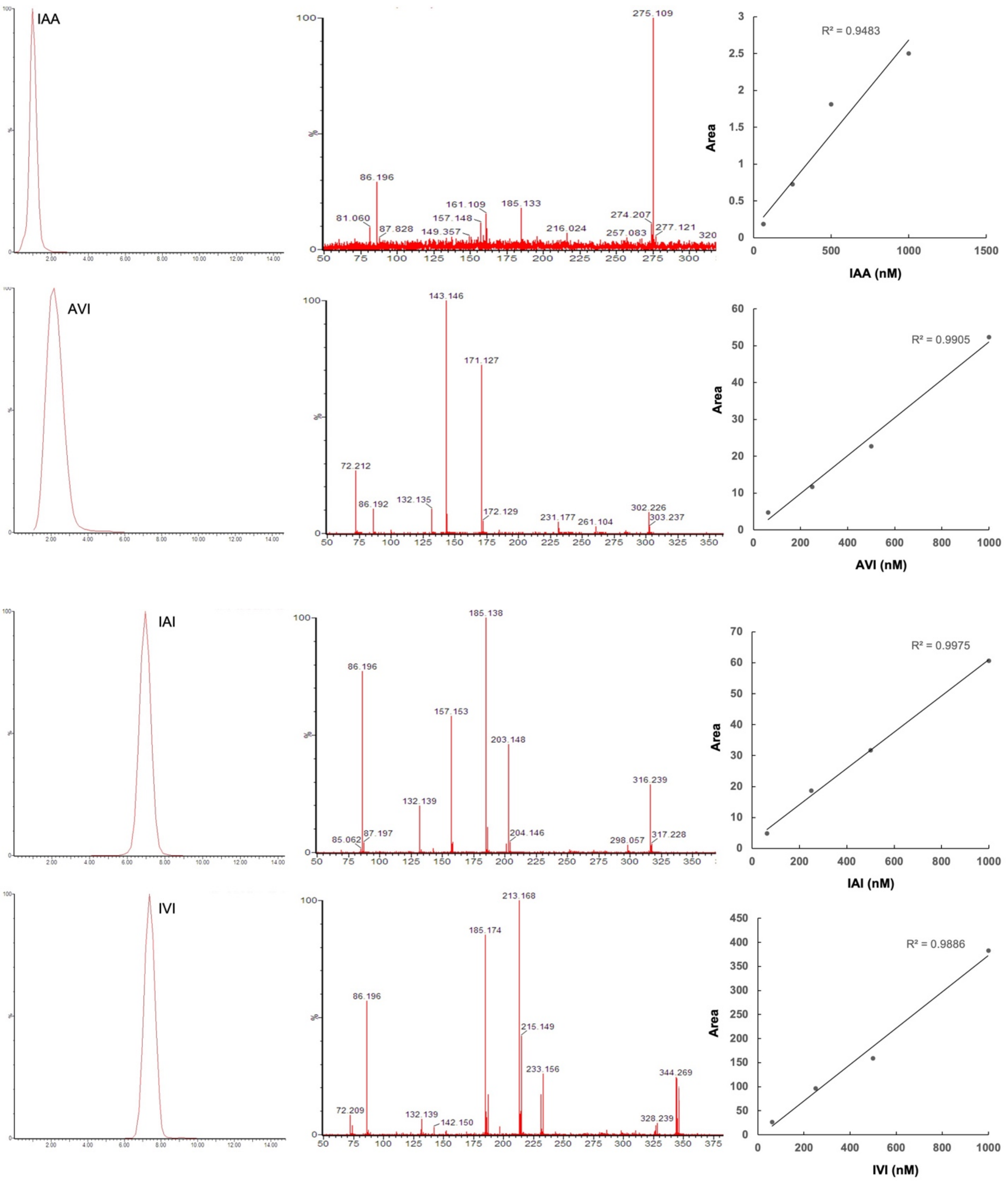

Figure S1, cont'd

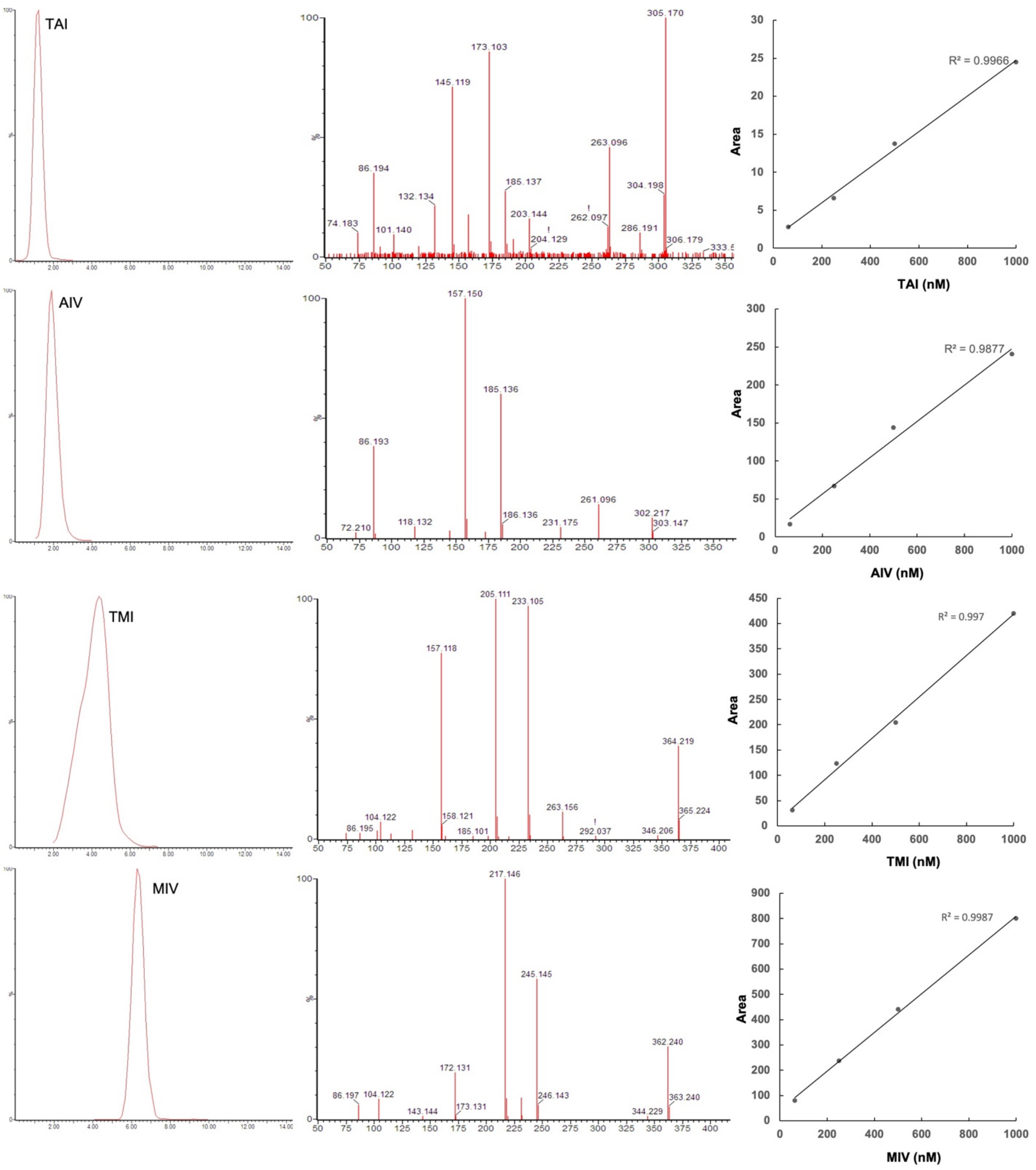

Figure S1, cont'd

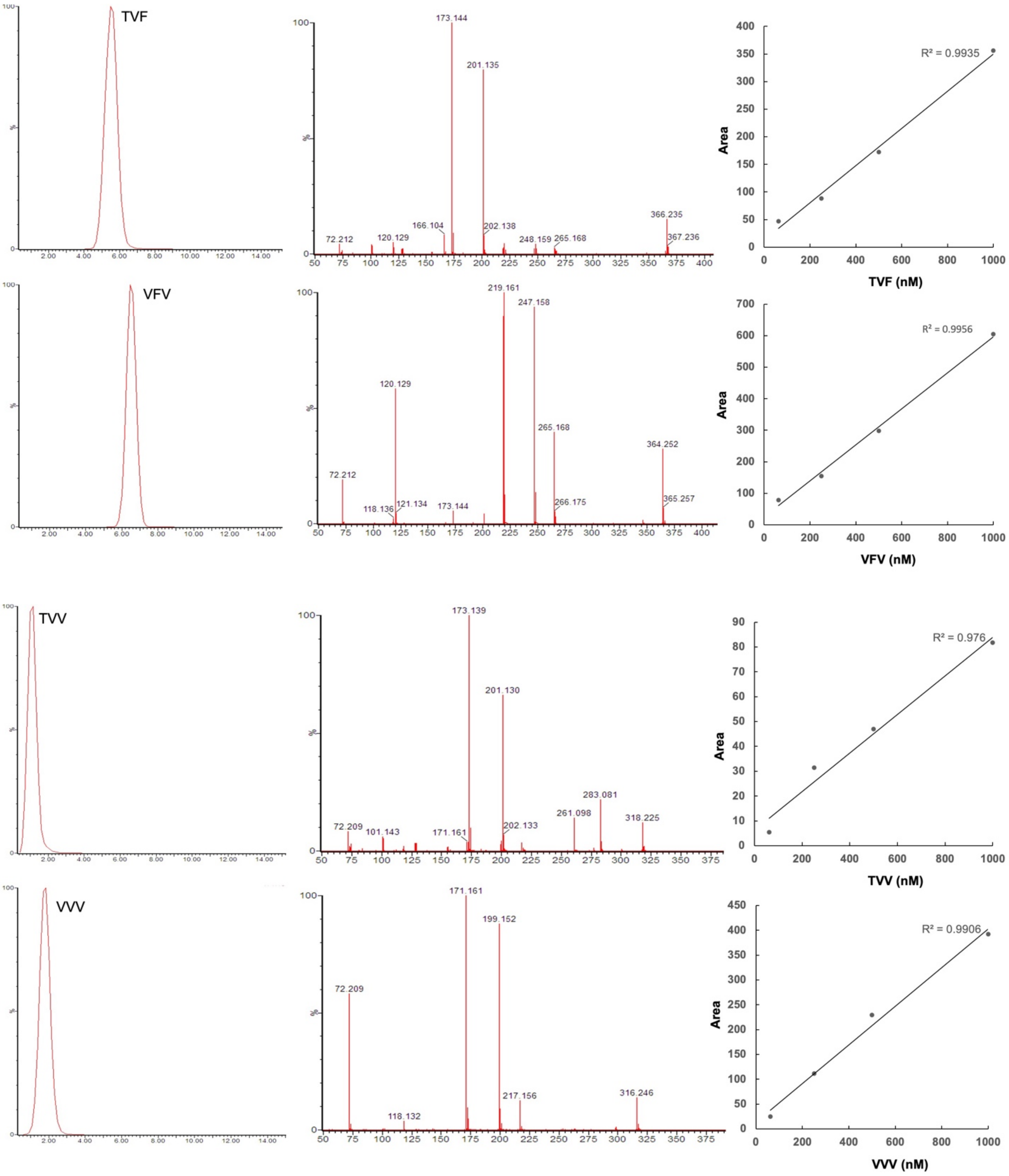

Figure S1, cont’d

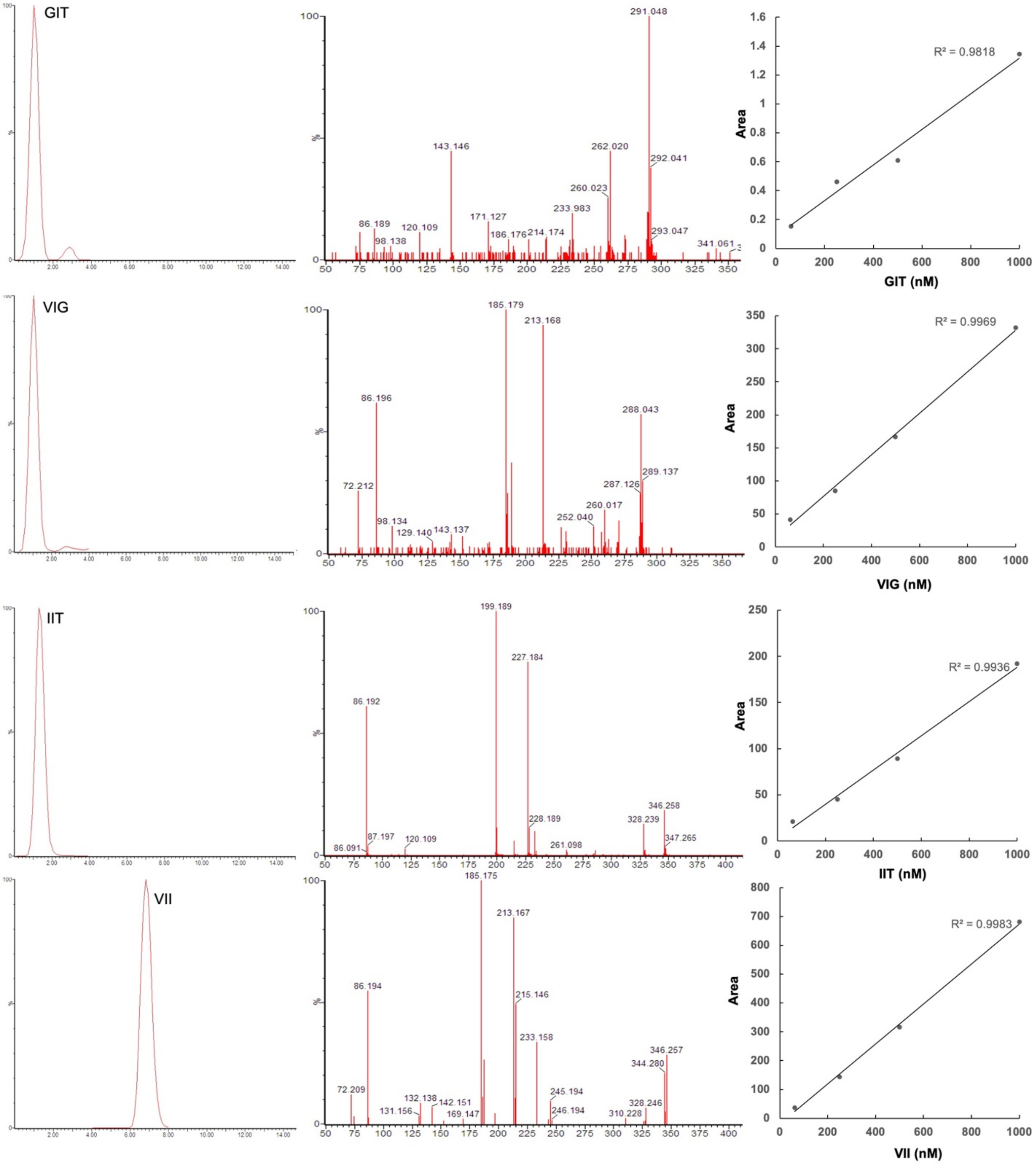

Figure S1, cont'd

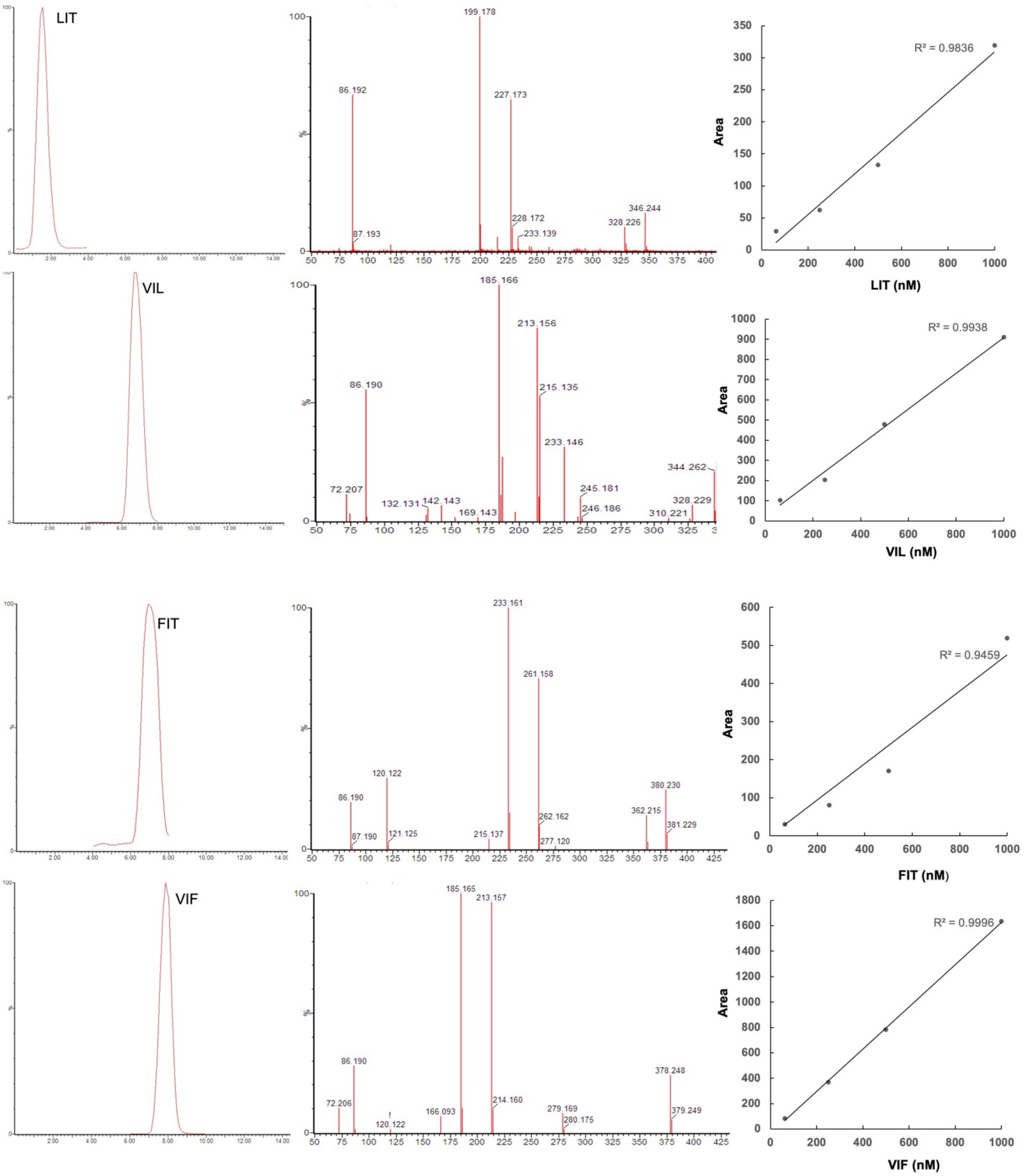

Figure S1. LC-MS/MS of all small-peptide standards predicted to be generated for all C100 substrates (WT and FAD mutants) after γ-secretase digestion of substrates. Chromatograms are selected ion plots of the three most abundant sequence-specific

product ions, selected with a 0.03 unit window. MS fragmentation of peptide standards are shown as inset to each chromatogram. Standard curves for all small peptides were generated by plotting of the resulting peak areas of ion plots against the small-peptide concentration.

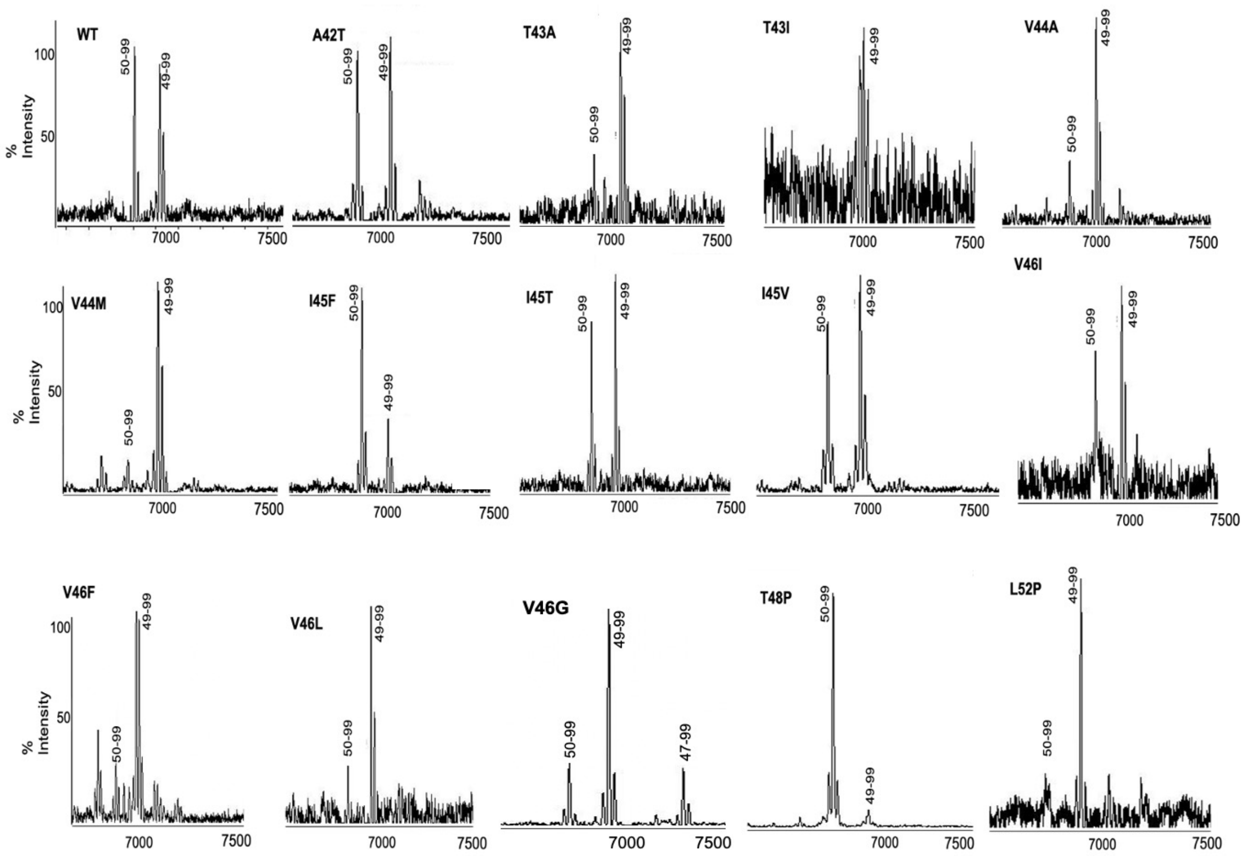

Figure S2. MALDI-TOF MS detection of AICD 50-99 and AICD 49-99 products derived from proteoliposome-based γ-secretase assay.

	% Cleavage of					
	Aβ49	Aβ48	Aβ46	Aβ45	Aβ43	Aβ42
**WT**	89	90	93	60	66	16
**A42T**	108	127	35	23	126	nd
**T43A**	82	69	63	73	101	2
**T43I**	54	48	104	98	69	12
**V44A**	66	40	102	71	86	30
**V44M**	55	41	100	41	82	17
**I45F**	119	104	8	61	80	6
**I45T**	14	69	nd	44	nd	nd
**I45V**	69	21	90	86	42	22
**V46I**	39	62	87	35	72	23
**V46G**	120	61	53	54	nd	22
**V46F**	121	30	53	38	96	35
**V46L**	84	36	42	49	105	30
**T48P**	17	59	3	75	nd	nd
**L52P**	174	6	nd	nd	nd	nd

Figure S3. Cleavage efficiency of Ab peptide proteolytic intermediates by γ-secretase, calculated as ([co-product of degradation] / [co-product of production]) x 100. Blue dotted line denotes cleavage efficiency from WT APP substrate. Nd, one or both of the cleavage products were not detected.
